# Maternal sociodemographic characteristics, early pregnancy behaviours, and livebirth outcomes as congenital heart defects risk factors - Northern Ireland 2010-2014

**DOI:** 10.1186/s12884-021-04223-4

**Published:** 2021-11-10

**Authors:** Hafi Saad, Marlene Sinclair, Brendan Bunting

**Affiliations:** 1 Maternal Fetal and Infant Research Centre, Ulster University, Jordanstown, UK; 2 School of Psychology, Ulster University, Coleraine, UK

**Keywords:** Congenital Heart Defects (CHD), Risk Factors, Population Based, Data Linkage, Cohort, Anonymised Data, Northern Ireland

## Abstract

**Background:**

Congenital Heart Defects (CHD) is the most commonly occurring congenital anomaly in Europe and a major paediatric health care concern. Investigations are needed to enable identification of CHD risk factors as studies have given conflicting results. This study aim was to identify maternal sociodemographic characteristics, behaviours, and birth outcomes as risk factors for CHD. This was a population based, data linkage cohort study using anonymised data from Northern Ireland (NI) covering the period 2010-2014. The study cohort composed of 94,067 live births with an outcome of 1162 cases of CHD using the International Statistical Classification of Diseases and Related Health Problems (ICD)-10 codes and based on the European Surveillance of Congenital Anomalies (EUROCAT) grouping system for CHD. CHD cases were obtained from the HeartSuite database (HSD) at the Royal Belfast Hospital for Sick Children (RBHSC), maternal data were extracted from the Northern Ireland Maternity System (NIMATS), and medication data were extracted from the Enhanced Prescribing Database (EPD). STATA version 14 was used for the statistical analysis in this study, Odds Ratio (OR), 95% Confident intervals (CI), P value, and logistic regression were used in the analysis. Ethical approval was granted from the National Health Service (NHS) Research Ethics Committee.

**Result:**

In this study, a number of potential risk factors were assessed for statistically significant association with CHD, however only certain risk factors demonstrated a statistically significant association with CHD which included: gestational age at first booking (AOR = 1.21; 95% CI = 1.04-1.41; P < 0.05), family history of CHD or congenital abnormalities and syndromes (AOR = 4.14; 95% CI = 2.47-6.96; P < 0.05), woman’s smoking in pregnancy (AOR = 1.22; 95% CI = 1.04-1.43; P < 0.05), preterm birth (AOR = 3.01; 95% CI = 2.44-3.01; P < 0.05), multiple births (AOR = 1.89; 95% CI = 1.58-2.60; P < 0.05), history of abortion (AOR = 1.12; 95% CI = 1.03-1.28; P < 0.05), small for gestational age (SGA) (AOR = 1.44; 95% CI = 1.22-1.78; P < 0.05), and low birth weight (LBW) (AOR = 3.10; 95% CI = 2.22-3.55; P < 0.05). Prescriptions and redemptions of antidiabetic (AOR = 2.68; 95% CI = 1.85-3.98; P < 0.05), antiepileptic (AOR = 1.77; 95% CI = 1.10-2.81; P < 0.05), and dihydrofolate reductase inhibitors (DHFRI) (AOR = 2.13; 95% CI = 1.17-5.85; P < 0.05) in early pregnancy also showed evidence of statistically significant association with CHD.

**Conclusion:**

The results of this study suggested that there are certain maternal sociodemographic characteristics, behaviours and birth outcomes that are statistically significantly associated with higher risk of CHD. Appropriate prevention policy to target groups with higher risk for CHD may help to reduce CHD prevalence. These results are important for policy makers, obstetricians, cardiologists, paediatricians, midwives and the public.

## Introduction

Congenital heart defects (CHD) is the most commonly occurring congenital anomaly in Europe [1], and one of the main concerns in paediatric health care which has a serious impact on infant mortality rates worldwide [2–4]. CHD impacts on health services as well as patients and their families [5–7], it represents a major global health problem and efforts should be continued to decrease the burden of the disease and to offer advice where possible, on reduction of risks of CHD.

Identifying the risk factors for CHD is of importance as intervening in relation to a particular risk may lead to prevention of CHD. Studies have been undertaken in the field of CHD risk factor research but have shown conflicting results [8]. Therefore, additional investigations are needed to enable accurate and robust identification of CHD risk factors [9] and to ensure that the required interventions are targeted more appropriately.

This study aimed to explore the impact of maternal sociodemographic characteristics and lifestyle behaviours as risk factors for CHD in Northern Ireland (NI) and to contribute to the current evidence on CHD to inform public health and social care policy.

NI is ideal for studying the risk factors for CHD as there is a single centre for foetal and paediatric cardiology in the Royal Belfast Hospital for Sick Children (RBHSC), and all children in NI with CHD are first seen at this centre. Administrative and clinical records of all pregnant women in NI and General Practitioner (GP) prescribing data are available centrally through the Honest Broker Service (HBS) [10] for (Health and Social Care) HSC sector in NI.

## Methodology

### Study design

This was a population-based cohort study based on secondary data analysis of linked datasets from HeartSuite Database (HSD), Northern Ireland Maternity System (NIMATS), and Enhanced Prescribing Database (EPD) (These datasets are described in another paper [11]).

### Study population and period

The study population includes all live births registered to women residing in NI during the period 2010-2014.

### Dataset description

For maternal sociodemographic characteristics and behaviours; risk factors were collected from details of the first booking visit from NIMATS database (92% of women gave this information in week 14 or less). Of the 96,233 pregnancies, 94,804 gave birth to cases without CHD (98.52%), and 1429 gave birth to CHD cases (1.48%). The births were to NI resident women, based on NI postcode and registered in the original NIMATS. For multiple pregnancies, only the baby with CHD was included; if there was no baby with CHD, only the most recent pregnancy was included, except where a mother had more than one baby with CHD, in which case both pregnancies were included, and except in the case of multiple births, when all were included.

Data from HSD: the CHD cases are major CHD cases recorded in HSD file based on EUROCAT grouping [12] using ICD10 codes containing a value between Q20.0 and Q26.9, except for Q25.0, Q25.6, or Q26. The sample includes only NI resident babies.

Medications prescribed and redeemed were obtained from EPD: an extract from the EPD, which contains detailed information in relation to NI prescriptions issued in primary care and redeemed by patients (based on British National Formulary (BNF) classification) was prepared by the HBS. This extract only includes data for NI-resident women registered in NIMATS for the period 2010-2014 and is only specific to each woman’s Exposure Window (EW), where EW = date of Last Menstrual Period (LMP) - 30 days and date of LMP + 90 days, using the date of each medication prescription. The EW begins 30 days prior to the first day of the woman’s LMP to enable medication prescriptions taken immediately before conception and possibly taken in early pregnancy to be included. This timing has been used in the design of other studies researching medication transfer in pregnancy [13–16].

The aetiology of CHD is apparent among babies with chromosomal and genetic syndromes, and among pregnant woman who are using vitamin A, anti-diabetic, or anti-epileptic medication, therefore these cases were excluded in the final file for analysis. This helps to increase the sensitivity of this study to assess the association with the other risk factors. However, before exclusion the associations between anti-diabetic or anti-epileptic medication and CHD were assessed. 222 chromosomal and genetic syndromes were excluded according to the following ICD10 codes Q90.0-Q93.0, Q96.0-Q99.9, Q44.71, Q61.90, Q74.84, Q75.1, Q75.4, Q75.81, Q87, Q93.6, and D82.1.

Another 1680 cases in which the pregnant woman redeemed a prescription for vitamin A, anti-diabetic, or anti-epileptic medication were excluded. The exclusions were based on BNF sections. Section 9.6.1 for vitamin A, sections 6.1.1 and 6.1.2 for anti-diabetic, and section 4.8 for anti-epileptic medication (https://openprescribing.net/bnf/). As the cause of stillbirth was not known in this study, 264 pregnancies which resulted in stillbirths were excluded.

**Fig. 1:**
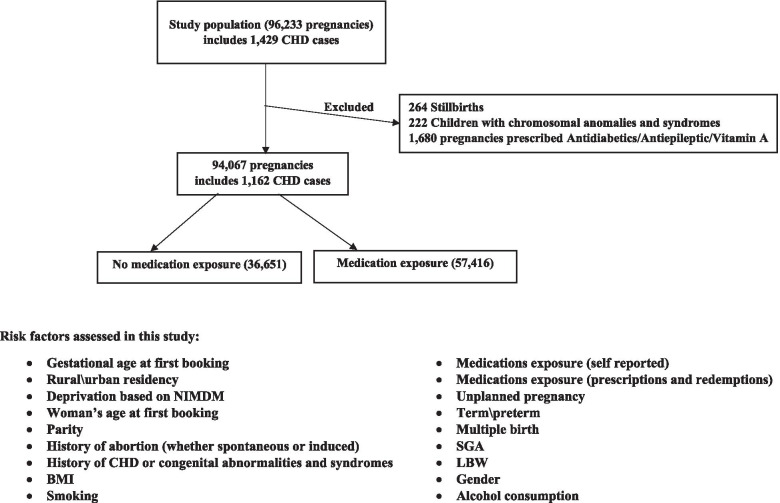
Flow of the data in the study population NI during the period 2010-2014, including risk factors assessed in the study. CHD=Congenital Heart Defects, BMI=Body Mass Index, SGA = Small for Gestational Age, LBW = Low Birth Weight, NIMDM = Northern Ireland Multiple Deprivation Measures

The final file therefore includes 94,067 pregnancies including 1162 CHD cases. The flow of data is shown in Fig. 1.

### Data linkage

During the study period there were 1605 CHD cases in HSD file and of these, 1429 CHD cases were able to be linked to NIMATS (HSD file contains only CHD cases, mothers in NIMATS which are not linked to HSD are assumed to be mothers of cases with no CHD) by HBS through deterministic linking using the infant Health and Care Numbers (INFANT_HCN). In the EPD extract there were 59,406 pregnancies which were linked successfully by the author (H.S.) to the NIMATS/HSD file using a common identifier (study_id) for both files.

### Statistical analysis

Odds Ratio (OR) with 95% confidence interval (CI) and P value were used as the association between exposure to specific risk factor and the CHD. Another frequently used measure of association reported in literature when publishing cohort study is risk ratio (RR) [17, 18]. Where outcomes are rare, as in the current case (typically <10%) the values associated with OR and RR are similar [19, 20]. As there is a binary outcome of interest (CHD or not) and a number of explanatory covariates (risk factors), logistic regression was used. To account for the possible clustering effects of multiple births, this covariate was included within the analysis.

The analysis was only performed when there were at least 3 CHD cases occurring after exposure or lack of exposure for each potential risk factor. Bivariate and multivariable analysis model parameters were estimated using maximum likelihood in STATA version 14.

### Missing data

Complete subject analysis was adopted and subjects with missing data in any potential risks were excluded from the analysis. The multivariable model is based on 90,079 pregnancies out of 94,067 pregnancies in the final file (approximately 96%). No imputation was conducted in this study and all percentages of missing values for each potential risk were presented.

### Covariates in the study

Those which have been examined for evidence of statistically significant association with CHD are gestational age at first booking, rural residency, pregnancy deprivation using Northern Ireland Multiple Deprivation Measures (NIMDM), woman’s age at booking, parity, family history of CHD or congenital abnormalities and syndromes, preterm birth, multiple births, history of abortion (whether spontaneous or induced), small for gestational age (SGA), low birth weight (LBW) (birth weight < 2500 g), gender, unplanned pregnancy, woman’s Body Mass Index (BMI), woman’s alcohol consumption, woman’s smoking in pregnancy, woman’s self-reported usage of folic acid (FA) and vitamins or any other group of medications, prescriptions and redemptions of FA and vitamin, mental health medication (MHM), and other medications.

## Results

Table 1 shows the association between the 18 potential risk factors assessed in this study and CHD using OR, 95% CI, and P value. Women who booked their first antenatal appointment after 14 weeks of pregnancy had an increased risk of their offspring developing CHD (P = 0.01). Family history of CHD or history of CHD and congenital abnormalities and syndromes showed strong evidence of statistically significant positive association with CHD (P < 0.01). The positive association with CHD also increased significantly in pregnant women who smoked (P < 0.05).

**Table 1:** Association between potential risk factors and CHD in NI (2010-2014) using OR, 95% CI, and P value (bivariate/multivariable analysis)

Covariate	No CHD	CHD	Total	% from total	Crude OR 95% CI	P value	Adjusted OR* 95% CI	P value
**1. Gestational age at first booking**								
≤14 weeks	85,126	1043	86,169	92				
>14 weeks	7779	119	7898	8	1.25 (1.03-1.51)	0.02	1.21 (1.04-1.41)	0.01
Missing	0	0	0	0				
Total	92,905	1162	94,067	100				
**2. Rural residency**								
Urban	59,637	746	60,383	64.19				
Rural	31,999	414	32,413	34.46	1.03 (0.92-1.17)	0.58	1.04 (0.92-1.18)	0.52
Missing	#	#	1271	1.35				
Total	#	#	94,067	100				
**3. Deprivation using NIMDM2010**								
Quintile 5 (Least deprived)	14,167	180	14,347	15.25				
Quintile 4	17,880	218	18,098	19.24	0.96 (0.79-1.17)	0.68	0.94 (0.77-1.15)	0.56
Quintile 3	18,971	259	19,230	20.44	1.07 (0.89-1.30)	0.46	1.03 (0.84-1.25)	0.38
Quintile 2	19,529	225	19,754	21.0	0.91 (0.74-1.10)	0.33	0.86 (0.70-1.05)	0.14
Quintile 1 (Most deprived)	21,157	279	21,436	22.79	1.04 (0.86-1.25)	0.70	0.97 (0.80-1.19)	0.58
Missing	#	#	1202	1.28				
Total	#	#	94,067					
**4. Parity**								
P0 (Nulliparous)	33,071	469	33,540	35.66				
P1	33,852	403	34,255	36.42	0.84 (0.73-0.96)	0.01	0.77 (0.70-0.88)	0.01
P2	16,888	173	17,061	18.14	0.72 (0.61-0.86)	<0.01	0.69 (0.59-0.79)	<0.01
P3	5932	64	5996	6.37	0.76 (0.59-0.99)	0.04	0.75 (0.57-0.99)	0.04
P4	1764	28	1792	1.91	1.12 (0.76-1.64)	0.57	1.01 (0.71-1.44)	0.41
≥ P5 (Grand multipara)	970	17	987	1.05	1.24 (0.76-2.01)	0.39	1.24 (0.76-2.01)	0.39
Missing	#	#	436	0.46				
Total	#	#	94,067					
**5. Woman’s age at first booking**								
25-29 years	25,968	342	26,310	27.97				
20-24 years	14,863	167	15,030	15.98	0.85 (0.71-1.32)	0.09	0.80 (0.66-0.98)	0.05
30-34 year	28,919	370	29,289	31.14	0.97 (0.84-1.13)	0.70	0.98 (0.85-1.15)	0.69
35-39 years	15,280	186	15,466	16.44	0.92 (0.77-1.11)	0.39	0.90 (0.75-1.09)	0.27
40+ years	3091	36	3127	3.32	0.88 (0.63-1.25)	0.49	0.84 (0.59-1.21)	0.36
<20 years	4738	58	4796	5.10	0.93 (0.70-1.23)	0.61	0.87 (0.64-1.17)	0.35
Missing	#	#	49	0.05				
Total	#	#	94,067					
**6. History of CHD or congenital abnormalities and syndromes**								
No history of CHD or congenital abnormalities and syndromes	81,641	841	82,482	87.68				
Only history of CHD	6310	237	6547	6.96	3.65 (3.15-4.22)	<0.01	3.62 (3.11-4.21)	<0.01
Only history of congenital abnormalities and syndromes	4593	68	4661	4.95	1.44 (1.12-1.84)	<0.01	1.28 (0.99-1.67)	0.05
History of CHD and congenital abnormalities and syndromes	361	16	377	0.40	4.3 (2.60-7.13)	<0.01	4.14 (2.47-6.96)	<0.01
Missing	0	0	0	0				
Total	92,905	1162	94,067					
**7. Woman’s smoking in pregnancy**								
No smoking	77,453	933	78,386	83.33				
Smoking	15,383	228	15,611	16.60	1.23 (1.06-1.42)	<0.01	1.22 (1.04-1.43)	0.02
Missing	#	#	70	0.07				
Total	#	#	94,067					
**8. Alcohol consumption**								
No alcohol consumption	91,910	1149	93,059	98.93				
Alcohol consumption	914	12	926	0.98	1.05 (0.59-1.86)	0.87	0.96 (0.53-1.76)	0.81
Missing	#	#	82	0.09				
Total	#	#	94,067					
**9. Woman’s BMI**								
Normal	44,395	541	44,936	47.77				
Overweight	26,041	329	26,370	28.03	1.04 (0.90-1.19)	0.61	1.04 (0.79-1.10)	0.59
Obese	15,027	186	15,213	16.17	1.02 (0.86-1.20)	0.86	1.01 (0.71-1.01)	0.73
Morbidly obese	1924	22	1946	2.07	0.94 (0.61-1.44)	0.77	0.77 (0.33-1.45)	0.59
Underweight	2033	31	2064	2.19	1.25 (0.87-1.80)	0.23	1.17 (0.69-1.55)	0.19
Missing	3485	53	3538	3.76				
Total	92,905	1162	94,067					
**10. Unplanned pregnancy**								
Planned	63,938	792	64,730	68.81				
Unplanned	26,535	330	26,865	28.56	1.00 (0.88-1.14)	0.95	1.01 (0.87-1.18)	0.87
Missing	2432	40	2472	2.63				
Total	92,905	1162	94,067					
**11. Woman’s self-reported usage of medication**								
None	5103	51	5154	5.48				
FA and vitamins	68,878	849	69,727	74.12	1.23 (0.93-1.64)	0.15	1.11 (0.85-1.55)	0.13
Other medications**	18,924	262	19,189	20.40	1.39 (1.02-1.87)	0.03	1.14 (1.01-1.66)	0.02
Missing	0	0	0	0				
Total	92,905	1162	94,067					
**12. Prescriptions and redemptions of medications in EPD**								
No redemptions	36,195	456	36,651	38.96				
FA and Vitamin	9883	120	10,003	10.63	0.96 (0.79-1.18)	0.72	0.89 (0.77-1.18)	0.61
DHFRI	#	#	#	#	2.56 (1.13-5.80)	0.02	2.13 (1.17-5.85)	0.01
MHM	6873	103	6976	7.42	1.19 (0.96-1.48)	0.11	1.33 (0.87-1.68)	0.10
Other medications***	#	#	#	#	0.95 (0.84-1.09)	0.52	0.98 (0.77-1.18)	0.42
Missing	0	0	0	0				
Total	92,905	1162	94,067					
**13. Preterm birth**								
≥ 37 weeks (Term)	86,240	943	87,183	92.68				
<37 weeks (Preterm)	6587	218	6805	7.23	3.03 (2.61-3.51)	<0.01	3.01 (2.44-3.01)	<0.01
Missing	#	#	79	0.08				
Total	#	#	94,067					
**14. Multiple birth**								
Single birth	89,732	1085	90,817	96.55				
Multiple birth	3173	77	3250	3.45	2.01 (1.59-2.54)	<0.01	1.89 (1.58-2.60)	<0.01
Missing	0	0	0					
Total	92,905	1162	94,067					
**15. History of previous miscarriage or termination of pregnancy**								
No history of abortion	67,230	803	68,033	72.32				
History of abortion	25,247	351	25,598	27.21	1.16 (1.03-1.32)	0.02	1.12 (1.03-1.28)	0.02
Missing	#	#	436	0.46				
Total	#	#	94,067					
**16. SGA**								
No SGA	83,513	991	84,504	89.83				
SGA	8964	163	9127	9.70	1.53 (1.30-1.81)	<0.01	1.44 (1.22-1.78)	<0.01
Missing	#	#	436	0.46				
Total	#	#	94,067					
**17. Low birth weight (<2500 g)**								
No low birth weight	87,552	971	88,523	94.11				
Low birth weight	3684	134	3818	4.06	3.28 (2.73-3.94)	<0.01	3.1 (2.22-3.55)	<0.01
Missing	1669	57	1726	1.83				
Total	92,905	1162	94,067					
**18. Gender**								
Male	47,460	609	48,069	51.10				
Female	45,427	553	45,980	48.88	0.95 (0.84-1.07)	0.37	0.89 (0.67-1.12)	0.32
Missing	18	0	18	0.02				
Total	92,905	1162	94,067					

# Disclosure control by the researcher for numbers less than 10, as per the process laid out by HBS

*SGA* small for gestational age, *MHM* mental health medication, *FA* folic acid, *DHFRI* Di hydro folate reductase inhibitors

*Adjusted OR for gestational age at first booking, urban/rural residency, deprivation using NIMDM, parity, woman’s age at first booking, unplanned pregnancy, history of CHD or congenital abnormalities and syndromes, woman’s BMI, woman’s smoking, alcohol consumption, woman’s self-reported usage of medication, preterm baby, multiple birth, prescription and redemption of medication, history of abortion, SGA, LBW, and gender

**other medications in NIMATS are any medication except FA and vitamins

***other medications in EPD are any medication that is not FA, DHFRI, or MHM

The multivariable model is based on 90,079 pregnancies out of 94,067 pregnancies in the final file (approximately 96%)

Women’s usage of medications in pregnancy (based on self-reported data taken from NIMATS), showed that taking other medications (other medications in NIMATS are any medication except FA and vitamins) had statistically positive association with CHD (P < 0.05). Medications from EPD are categorised into non-medication, FA and vitamin, dihydrofolate reductase inhibitors (DHFRI), MHM, and other medications (other medications in EPD are any medication that is not FA, DHFRI, or MHM). Using the EPD file showed that prescription and redemption of MHM has no significant association with CHD (P = 0.40). In contrast, DHFRI have shown a significantly increased risk of developing CHD (P < 0.05). However, FA supplement alone showed no significant protective association with CHD (P > 0.05).

Before excluding pregnancies which were exposed to antidiabetic and antiepileptic medications, their association with CHD was assessed, and both antidiabetic and antiepileptic medications showed statistically significant association with CHD with (AOR = 2.68; 95% CI = 1.85-3.98; P < 0.01) and (AOR = 1.77; 95% CI = 1.10-2.81; P < 0.02), respectively.

Other potential risk factors related to the baby were examined, and data showed evidence of positive association with CHD and preterm birth (P < 0.01), LBW (birth weight < 2500 g) (P < 0.01), SGA (P < 0.01), and multiple births (P < 0.01). There was strong evidence of a positive association between. history of abortion and CHD (P < 0.05).

## Discussion

Women who booked their first antenatal appointment after 14 weeks of pregnancy had an increased risk of their offspring developing CHD, this may be because provision of antenatal care (ANC) before 14 weeks allows guidance to be provided at an early stage on modifiable lifestyle risks such as smoking, alcohol consumption and using certain teratogens. Early ANC also captures DM and could enhance blood glycaemic control. It is therefore crucial for ensuring optimal care and good maternal and foetal health [21]. Late booking (which delays ANC) may delay dealing with the risks outlined above, most of which have shown an increased association with CHD in offspring. The study suggests that health service planning should investigate the reasons behind late first booking appointments and ensure a policy to reduce these due to the association with CHD and other potential adverse outcomes for both babies and pregnancies.

There was no higher risk of developing CHD among infants whose mothers lived in rural areas in comparison to urban areas. The literature showed conflicting results regarding the association between rural or urban residency and CHD in offspring [22, 23]. Being categorised in the most deprived group based on NIMDM as a proxy of SES was not shown to have a statistically significant association with CHD. And there is no evidence that differences in SES (based on area level measures) affect the occurrence of CHD in NI. However, the findings of this study may be consistent with a finding from a meta-analysis, which showed no clear relationship between SES and CHD in developed countries [24].

Parity was not shown to have a statistically significant association with CHD, meta-analysis showed no statistically significant association between parity (comparing highest to lowest) and CHD in studies conducted after 2010 [25] and this is consistent with the findings of this study.

Regarding the woman’s age at first booking, no statistically significant association was shown between older women (>40 years) and CHD. These results are challenging as they do not support earlier research [26, 27]. However, they do support more recent research reporting a lack of positive association between CHD and advanced age [28]. This data may alleviate anxiety amongst older mothers and reduce the number of referrals for early echocardiography to detect CHD that are primarily based on older age. It may also reassure older women that their age in itself does not confer additional risk of CHD, and it may support the belief that women in this age group take extra care during their pregnancy to protect their baby [28].

There was evidence in this study of a statistically significant positive association between maternal smoking and CHD. This finding suggests that population-based prevention strategies targeting smoking cessation in pregnancy should highlight this message. A decrease in maternal smoking during pregnancy could result in decreased risk of CHD. The positive association of maternal smoking with CHD found in this study is consistent with findings from National Birth Defects Prevention Study (NBDPS) [29]. The NBDPS database identified an increased risk of developing specific subtypes of CHD in offspring of pregnant women who smoked [29], and a meta-analysis showed a positive association between maternal smoking during pregnancy and risk of CHD [30]. The mechanisms through which smoking may cause CHD are still to be clearly identified. It has been postulated that maternal smoking has harmful effects on the development of the foetus; carbon monoxide and nicotine induce hypoxia and reduce the supply of essential nutrients to the embryo [31, 32]. Additionally, common components of cigarette smoke such as polycyclic aromatic hydrocarbons are suspected teratogens in laboratory tests on humans and animals [33, 34]. From a public health policy perspective, finding from this study and others may fuel the need to ring-fence funding for smoking cessation prenatally and antenatally.

No statistically significant association with CHD was shown for alcohol consumption in this study. While this result is important it should not be interpreted as evidence that maternal drinking is safe, as it is known that prenatal exposure to low levels of alcohol, such as a single unit, can result in foetal alcohol syndrome (FAS) [35]. Nevertheless, no significant association between alcohol consumption in pregnancy and CHD was found in two recent meta-studies with a large sample size; these findings are consistent with those of the current study [36, 37].

Previous meta-analysis demonstrated that offspring of women with a history of abortion (whether spontaneous or induced) had a higher risk of developing CHD [38], a finding which was repeated in this study. While the specific biological mechanism which underlies the link between a history of previous abortion and the risk of CHD is still unclear, it has been suggested that history of abortion can cause mental stress to mothers [39], and literature [40, 41] found that mothers who were exposed to stress during pregnancy might have an increased risk of CHD in their offspring.

Family history of CHD or congenital abnormalities and syndromes is associated with increased risk of CHD. This finding has relevance for any preventative policy for CHD, such as pre-conceptional counselling for parents with a family history of CHD. Midwives routinely collect this data and identify risk factors for congenital anomalies. However, evidence from literature and this study may lead to clinical policy developments resulting in more screening referrals for foetal cardiology at booking and more midwifery input to counselling mothers prior to tests. The positive association of family history of CHD or congenital abnormalities and syndromes and CHD found in this study is consistent with those of other studies in Denmark, Canada, and USA [42, 43].

The study has shown statistically positive association between CHD and prescription and redemption of FA antagonists group of medications (including antiepileptic and DHFRI) in early pregnancy. Clinicians in particular will be interested in this result as it may help to guide their prescription practice and midwives booking mothers into the antenatal clinic need to be alerted to this risk factor. This result is consistent with the findings from previous studies that assessed the association between FA antagonists and CHD, which showed that usage of FA antagonists (either DHFRI or antiepileptic) to be associated with an increased risk of CHD [44–46].

The pharmacological plausibility of this positive association confirms the importance of this finding at public health policy level. Impaired folate metabolism has been found to affect cardiac neural crest cell formation and migration and it has been suggested that this might interfere with heart development [47–50]. Impaired FA transport has led to extensive death of apoptotic cells in the developing heart [51].

Unplanned pregnancy has been shown to have no significant positive association with CHD. This is an important finding, as there is a scarcity of studies that address this question. It should be noted, however, that unplanned pregnancy may be more likely to be associated with certain behaviour in early pregnancy such as smoking, lower educational attainment [52], and outcomes such as preterm labour [53], all of which are associated with a higher risk of CHD.

FA supplementation showed no protective effect for CHD. This finding, which is based on data from two different sources (NIMATS and EPD), is surprising as most previous research has shown a protective effect for FA [54] and there is a plausible biological mechanism to justify this protective effect. This finding may call into question any policy which aims to reduce CHD prevalence by imposing population based folic acid fortification in NI. However, this lack of protective effect should be received with caution; as suggested by Hobbs et al. [55], the absence of a protective association between FA and CHD cannot be asserted without further analysis of relevant environmental and genetic factors. Furthermore, the finding that FA antagonists (DHFRI and antiepileptics) are associated with increased risk of CHD may also indicate the importance of FA in CHD causation. The finding that FA had no protective effect also could be due to the misclassification of exposure, in that the level of FA required for individuals had already been attained; molecular techniques are needed to test this theory. However, it is possible that the finding could reflect a true absence of protective effect, as in fact has been shown by a number of studies that have found no significant protective effect of FA supplementation on CHD [56, 57].

This study showed no statistically significant association between maternal obesity and risk of CHD. However, it must be noted that this finding conflicts with previous literature in which obese pregnant women were found to have a significantly increased risk of CHD [58–60]. It is important to note that the mechanisms which explain the relationship between maternal obesity and CHD are unclear, although there is an argument that women who are obese may have Diabetes Mellitus (DM), which is a known risk factor for CHD [61]. The findings of this study support this argument as all DM cases were excluded from the analysis allowing the independent assessment of obesity as a risk factor for CHD.

In this study, certain factors are categorised as factors related to the child birth outcome, these were: SGA; multiple births; LBW; whether term or preterm; and gender. It was interesting to note that there had been statistically significant association between all those factors (except gender) and CHD. The literature supports these findings and shows that there are significant differences in the occurrence of CHD among SGA [62, 63], multiple births [64];, LBW [65], or preterm birth [63, 66, 67]. Multiple birth status potentially affects intrauterine growth and carries a higher risk of premature birth [68]. The mechanism behind why these groups carries a higher risk of having an infant with CHD is not clear. These groups are important to be aware of as they may need to be considered in the implementation of any preventative policy in relation to CHD. Given that some babies are more at risk of having CHD than others, considering these categories is essential to facilitate early detection (via appropriate diagnostic procedures) and appropriate intervention. From a clinical perspective, many CHDs are not diagnosed antenatally and are identified at birth or shortly thereafter. Early diagnosis is important in providing an opportunity for early treatment and the prevention of disabilities and death [69], therefore assessing risk factors and clinical decision making are critical factors to be taken into consideration when formulating CHD policies. This study showed no significant association between gender and CHD, and the literature on gender has shown conflicting results regarding its association with CHD [43, 70].

It is also crucial to comment on the finding that antidiabetic medication prescription during pregnancy is associated with CHD. This finding is highly significant as it suggests that putting women who have DM at the heart of any preventative policy for CHD may be useful. This result aligns with the current literature, which has shown positive association between pre-existing diabetes and CHD [61, 71–74]. The possible causal mechanism for this positive association is poorly understood but the main hypothesis clarifying the positive association between maternal DM and CHD in offspring is that a teratogenic effect on the developing heart may be caused by excess glucose [75]. Excess glucose may lead to epigenetic changes that would affect gene expression in the developing embryo [76].

The control of blood glucose in pregnancy could be one of the preventative measures for the development of CHD [77]. Blood glucose control requires regular blood check, diet control, exercise, and subsequent diabetic medication adjustment. Antidiabetic medication prescribed in this study included oral hypoglycaemic medication which may lack efficiency to overcome insulin resistance in pregnancies [78] and hence does not control for blood glucose level. According to the American Diabetes Association guidelines, diabetic women taking oral hypoglycemic medication might be at risk of treatment failure and they should switch to insulin when they are pregnant [79]. It should also be mentioned that there is a signal in the literature which showed that oral hypoglycemic medication such as metaformin might be associated with increased risk of CHD [80]. In addition, although women were prescribed antidiabetic medication, its prescription in this study might not reflect usage of antidiabetic medications (or any other medications) and literature has shown that there is discontinuation of oral antidiabetic medicines during pregnancy among large number of pregnant women [81, 82].

Most of the study covariates did have 0% missing values, and a few of them showed less than 4% missing values, which gives credibility to the study results (Table 1). The study provides an overview of the profile of pregnant women in NI which is useful for researchers. For example, it showed that 65% of pregnant women in NI live in urban areas, 15.6% smoke, and 64.7% have a planned pregnancy.

It should be noted that the livebirth prevalence of CHD in this data is higher than others in Europe. This may be due to complete ascertainment of both early and later diagnosed cases in the paediatric cardiology database used in this study as well as the fact that during the study period NI was one of the few European countries where termination of pregnancy for fetal anomaly was illegal.

## Strengths and limitations

This is the first population based study to use big data from linked administrative and clinical databases that cover the entire population in NI during the period 2010-2014 to identify risk factors for CHD, with a large sample size of 94,067 pregnancies and 1162 CHD cases. Different risk factors for developing CHD related to maternal sociodemographic characteristics, behaviours and birth outcomes were assessed and the study was able to adjust for several significant covariates. CHD diagnoses in this study were obtained from RBHSC which is the only centre in NI in which cases are examined by echocardiograph and diagnosed by paediatric cardiologists. Two studies using UK data reported good positive predictive values for ICD-10 codes (PPVs ≥90%) [83, 84]. There was high ascertainment for CHD diagnosis, all HSD cases underwent echocardiography by paediatric cardiologists using the same echo machines and following the same diagnostic criteria, thereby limiting the chance of case misclassification. Most of the study covariates have a low percentage of missing values which suggests that the data used is high quality. The study provides an overview of the profile of pregnant women in NI which is useful for researchers.

Recall bias has been avoided in this study by using prospectively collected data, as recall bias is expected when exposure data is collected retrospectively [85]. Retrospectively collected data with questionnaires may also involve interviewer bias which is also avoided in this study. However, linkage between CHD cases from HSD to NIMATS was not able to link 10.9% of the CHD cases and the exclusion of those cases from the analysis may have led to under-ascertainment of exposures or outcomes, and might have led to selection bias. Women who moved later in pregnancy and gave birth in NI are all included in the study, however there is no data on women and their births outcome for those who gave birth outside NI during the study period.

However, the study results are as precise as possible using available data and rigid inclusion and exclusion criteria and specifying the importance of the first trimester in highlighting any temporal relationship between potential risk and outcome, which would need to be considered in any future discussion of causation. Caution should be taken when applying the results of this study to other populations due to differences between populations, study period, and methodologies.

It should be noted that this an association rather than causation study, in future studies causation might be assessed more robustly inferred by the introduction of graphical models based on Directed Acyclic Graph (DAG) and using an epidemiological framework such as Bradford-Hill criteria to assess possible cause and effect.

The risk for CHD measured in this study is based on live birth estimates and no estimation was calculated based on stillbirths and termination of pregnancy, and this is one of the study limitations. Ideally, all foetuses with stillbirths and termination of pregnancy including termination of pregnancy for foetal anomalies (TOPFA) should be included, specifying those who have CHD. TOPFA could not be included as TOPFA was not permitted in NI unless: “a woman’s life is at risk or where there is a risk of a serious and adverse long term or permanent effect on her physical or mental health” [86].

Although accurate information about TOPFA in NI is not available, reports covering the period 2010-2014 have shown that some pregnant women travelled from NI to England, Scotland or Wales to have an abortion, including TOPFA. There were 4652 abortions performed in England and Wales on women who had travelled from NI during the period 2010-2014 [87]. It is not known how many of those cases have CHD but exclusion of these cases from the analysis may have affected the results. Stillbirths were not included in the study as HSD and NIMATS do not include information about stillbirth cases which are diagnosed as CHD (This practice requires post-mortem for all stillbirths, and this was not the practice in NI during the study period). However, excluding stillbirths from the current study would be expected to have a minimal effect as stillbirth occurs in approximately 264 pregnancies which represents 0.27% of all pregnancies in this study, and congenital abnormalities including CHD account for only 12-14% of stillbirths [88, 89]. The study estimation does not consider pregnancies which end in miscarriage or ectopic pregnancy. The ectopic pregnancy rate in the UK is 11 per 1000 [90] and 1 out of 4 pregnancies ends with miscarriage [91]. However, it is not common practice to include them in studies as it is difficult to identify the cause of miscarriage or whether the foetus in an ectopic pregnancy had a heart problem or not.

There is always a possibility of exposure misclassification in relation to a number of risk factors assessed in this study. For example, pregnant women tend to underestimate their smoking behaviour when self-reporting [92]. Moreover, the study did not examine the factors which may affect total smoking exposure such as environmental tobacco smoke. Future research using serum cotinine measurement to validate smoking risk will give more accurate information regarding the association between smoking in pregnancy and CHD.

Residual confounding by confounders such as ethnicity [93], certain environmental exposure [94], method of conception [95], and preeclampsia [96] may still be present, but the association between the potential residual confounders and CHD, must be strong and specific for CHD to have the potential to alter the findings of this study. In this study, several risks were assessed simultaneously leading to multiple testing issues and potential chance findings. No adjustment was made for multiple testing in this study as doing so may cause significant clinical associations to be lost. This approach has been adopted in other studies [12, 97].

Confounding by indication is one of the most important limitations of this study, in which individuals who were exposed to drugs may have experienced a contrary outcome (i.e. CHD) because they are more ill than others. Whether or not the pregnant women took the drugs as prescribed is not confirmed, as redemption of prescription does not necessarily indicate the medication was taken. There is therefore a risk of exposure misclassification, possibly leading to drug use overestimation. This has been considered in the study design in which the exposure period goes back to only one month before LMP in line with the literature which suggested that: “prescriptions given in the period prior to 1 month before the LMP would have resulted in a greater percentage of women who do not use medication during pregnancy” [98].

## Conclusion

The study provides data on the maternal sociodemographic characteristics, behaviours and birth outcomes as risk factors for CHD in NI during the period 2010-2014, which is important for public health policy, maternity service providers, researchers, clinicians, and the public.

The identified association and possible causation should be understood and discussed on logical plausibility, and within the overall context of the available evidence to promote CHD prevention. Potential risk factors which did not show positive significant association with CHD in this study should always be understood within the same context. The study has strengths but it has limitations which may also need to be addressed in future studies which involve linking big databases. Other risk factors which are not found in the study data and hence were not assessed should be assessed in future studies.

## Data Availability

The data that support the findings of this study are included at aggregate level in this manuscript. Individual data were available/accessed in an anonymised format in the safe haven in HBS under license for the current study. Due to the fact that individuals could be identified from a combination of variables, restrictions apply to the availability of these data publicly. For any query/request about this data please contact Dr. Hafi Saad (H-saad@uster.ac.uk).
